# Survey data on students’ online shopping behaviour: A focus on selected university students in Indonesia

**DOI:** 10.1016/j.dib.2019.105073

**Published:** 2020-01-02

**Authors:** Heri Kuswanto, Widyan Bima Hadi Pratama, Imam Safawi Ahmad

**Affiliations:** Institut Teknologi Sepuluh Nopember (ITS), Surabaya, Indonesia

**Keywords:** Online, Students, Cluster, Hierarchy

## Abstract

The data presented in this paper is used to examine the factors influencing students' online shopping behaviour and to identify the students' segmentation on the important factors. The survey was conducted in the Institut Teknologi Sepuluh Nopember (ITS) Surabaya, the biggest science and technology university in East Indonesia, with multicultural and diverse socio-economic students' backgrounds. The total number of population is 20448 students. Using Yamane's formula, a sample size of 393 students was surveyed online, and 83 of them experienced doing online shopping. A quantitative method with a descriptive research design was adopted to explore insights in the data related to the objective of the research. The survey data were analyzed by linear regression and hierarchical clustering. The conceptual framework of the variables are given, and reliability and validity have been confirmed. Data were analyzed with MINITAB and SPSS software.

**Table:** Specifications Table

Subject area	Business Management and Statistics
More specific subject area	Business Management: Marketing and Strategy Management
Type of data	The raw data is available in SPSS format (sav). The analyzed data in this article are provided in tables and figures.
How data was acquired	Online survey through questionnaire. The questionnaire is included in this article.
Data format	Raw, analyzed, descriptive and inferential statistical data
Experimental factors	Samples were collected from university students in Surabaya, Indonesia. The questionnaire assessing their online shopping behaviour was made.
Experimental features	University students are the largest potential market of online shopping in Indonesia
Data source location	Surabaya, Indonesia
Data accessibility	Data are included in this article.
Related research article	M. Zendehdel, L.H. Paim, S. B. Osman. Students' online purchasing behaviour in Malaysia: Understanding online shopping attitude, Cogent Business & Management, 2(1), 2015, 1–13 [1]

**Table:** 

**Value of the Data** • The dataset provides a significant contribution on capturing information about the students preference and behaviour in online shopping • The dataset can be used to understand the market segmentation for online shopping in Indonesia, which is a fundamental and crucial information for online shopping providers • The information in the dataset about factors influencing shopping behaviour can be used as a reference by online shopping providers (online marketplace) to improve their business strategy in order to attract a broader market • The dataset can be further analyzed using more advance statistical analysis involving latent variables such as Structural Equation Modelling to generate more insight about direct and indirect factors influencing student's behaviour • The dataset can be used as the basis to develop further experiments applying resampling methods due to small sample data provided.

## 1 Data

As shown in Table 1, the survey was administered to 393 students representing the sample size used in the selected university (ITS). Among these numbers, 83 (21%) of them indicated that they have experience in doing online shopping. Meanwhile 310 (79%) respondents had no experience with online shopping. The analyzed data in this paper involved only information collected from respondents with experience of doing online shopping.

**Table 1:** Analysis of general response dealing with experience in online shopping.

Experienced on online shopping	Number	Percentage
Yes	83	21%
No	310	79%
Total	393	100%

Table 2 shows the distribution of the respondents based on their semester level. We see that the majority (86.8%) of the respondents were in the 5th and 7th semester. The rest (13.2%) were students in the 1st, 3rd and 4th semester.

**Table 2:** Descriptive statistics on respondents’ semester level.

Respondents by semester level	Number	Percentage
1	3	3.6%
3	7	8.4%
4	1	1.2%
5	36	43.4%
7	36	43.4%
Total	83	100%

**Fig. 1:**
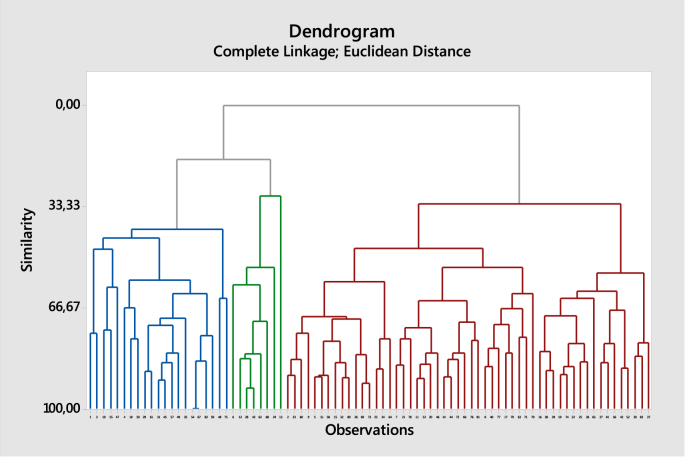
Dendogram of hierarchical clustering.

Table 3 describes the respondents’ responses from those 83 students, where 41% of them are male students and 59% are female students, as shown in Fig. 1. It basically shows that female students did shopping more than male students.

**Table 3:** Percentage distibution of gender of the surveyed students.

Gender	Number	Percentage
Male	34	41%
Female	49	59%
Total	83	100%

Table 4 shows that most (58%) of the respondents did online shopping an average of one time in a month, 30% did online shopping two times in a month and the rest (12%) did more than two transactions per month.

**Table 4:** Average frequency of online shopping within a month.

Frequency of online shopping in a month	Number	Percentage
One time	10	12%
Two times	48	57.8%
More than two times	25	30.2%
Total	83	100%

Table 5 presents the statistics of the respondents in more detail based on gender. We see that female students did shopping more than male students did. Based on the favourite online marketplace, the male students choose Tokopedia, Lazada and Bukalapak while female students mostly shopped at Sophee.

**Table 5:** Descriptive statistics based on gender.

Variable		Gender	
		Male	Female
Frequency of online shopping in a month	1 x	52%	48%
	2 x	10%	42%
	>2 x	8%	13%
Favourite online marketplace	Tokopedia	23%	8%
	Lazada	19%	6%
	Bukalapak	15%	0%
	OLX	2%	0%
	Shopee	4%	58%
	Zalora	0%	6%
	Berrybenka	0%	4%
	Others	8%	19%
Range of product price	<100000	6%	21%
	100000–200000	17%	46%
	200001–300000	23%	23%
	300001–500000	10%	10%
	500001–1000000	10%	2%
	>1000000	4%	0%
Product category	Accesories	13%	6%
	Electronics	29%	4%
	Fashion	19%	63%
	Hobby	8%	6%
	Household needs	2%	0%
	Skincare	0%	19%
	Voucher	0%	4%

Table 5 also revealed that the students spent mostly about 100000 IDR to 200000 IDR (the current exchange rate is 1 USD equivalent to about 14500 IDR). The male students mostly bought electronics (29%) while female students mostly purchased fashion (63%).

**Fig. 2:**
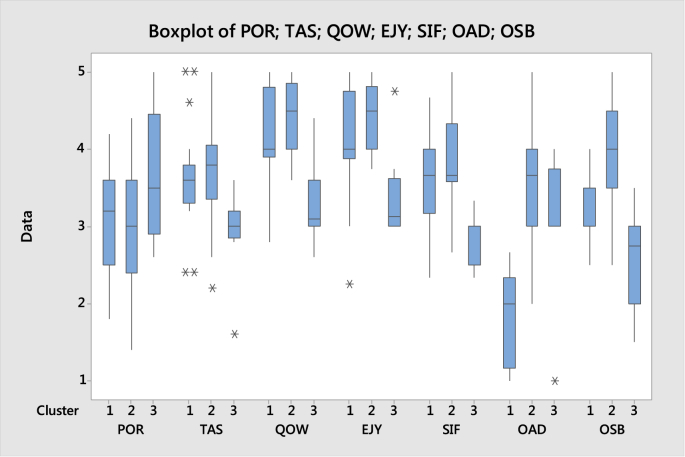
Boxplot of cluster characteristics for each attribute.

Based on the dataset, we can perform cluster analysis to identify the segmentation of the students. Fig. 1 depicts a dendogram created by using complete linkage with Euclidean distance measure. It provides cluster members depending on the number of clusters. Fig. 2 shows of boxplots of the segments assuming that we perform three clusters. In most cases, cluster 1 and cluster 2 relatively have similar characteristics (see also Table 6). Therefore, there might be only two clusters of students with significantly different characterstics. This fact is supported by the summary statistics in Table 3.

**Table 6:** Descriptive statistics of each cluster in each attribute.

Variable	Cluster	N	Mean	StDev	Minimum	Q1	Median	Q3	Maximum
POR	1	21	3.076	0.680	1.800	2.500	3.200	3.600	4.200
	2	54	3.037	0.735	1.400	2.400	3.000	3.600	4.400
	3	8	3.650	0.840	2.600	2.900	3.500	4.450	5.000

TAS	1	21	3.610	0.662	2.400	3.300	3.600	3.800	5.000
	2	54	3.744	0.642	2.200	3.350	3.800	4.050	5.000
	3	8	2.925	0.585	1.600	2.850	3.000	3.200	3.600

QOW	1	21	4.210	0.542	2.800	3.900	4.000	4.800	5.000
	2	54	4.444	0.437	3.600	4.000	4.500	4.850	5.000
	3	8	3.300	0.555	2.600	3.000	3.100	3.600	4.400

EJY	1	21	4.131	0.692	2.250	3.875	4.000	4.750	5.000
	2	54	4.476	0.412	3.750	4.000	4.500	4.812	5.000
	3	8	3.375	0.612	3.000	3.000	3.125	3.625	4.750

SIF	1	21	3.603	0.704	2.333	3.167	3.667	4.000	4.667
	2	54	3.808	0.520	2.666	3.583	3.666	4.333	5.000
	3	8	2.875	0.354	2.333	2.500	3.000	3.000	3.333

OAD	1	21	1.857	0.563	1.000	1.167	2.000	2.333	2.667
	2	54	3.586	0.700	2.000	3.000	3.666	4.000	5.000
	3	8	3.000	0.926	1.000	3.000	3.000	3.750	4.000

OSB	1	21	3.285	0.435	2.500	3.000	3.500	3.500	4.000
	2	54	4.037	0.678	2.500	3.500	4.000	4.500	5.000
	3	8	2.563	0.678	1.500	2.000	2.750	3.000	3.500

Table 7 presents the output of multiple linear regression analysis to investigate the factors influencing the online shopping behaviour. The hypothesis to be tested is as follow:

**Table 7:** Anaysis of variance.

Source	DF	Adj SS	Adj MS	F-Value	P-Value
Regression	6	24.3462	4.05770	11.35	0.000
POR	1	1.9412	1.94116	5.43	0.022
TAS	1	0.0384	0.03838	0.11	0.744
QOW	1	0.6563	0.65634	1.84	0.179
EJY	1	1.8530	1.85298	5.18	0.026
SIF	1	1.6500	1.65000	4.62	0.035
OAD	1	2.1992	2.19916	6.15	0.015
Error	76	27.1719	0.35752		
Lack-of-Fit	75	27.1719	0.36229	*	*
Pure Error	1	0.0000	0.00000		
Total	82	51.5181			

Ho: There are no variables influencing online shopping behaviour.

We see that the ANOVA produces P-value of the regression = 0.000, which is less than 0.05 significant level. This leads to the rejection of the null hypothesis, meaning that at least one of the predictors significantly influences the purchasing behaviour. The R-square is 47.26%, meaning that the predictors have an effect of 47.26% on onine shopping behaviour.

The coefficients in Table 8 show the individual effect of each variable. We see that the P-values of POR, EJY, SIF and OAD are less than 0.05 significant level. This means that the purchasing behaviour is significantly infuenced by the perception of risk (POR), enjoyment (EJY), social influence (SIF) and online advertisment (OAD). Meamwhile, two other variables, i.e. trust and security (TAS) and quality of website (QOW), did not significantly influence the online shopping behaviour (see Table 9).

**Table 8:** R-square of the regression.

S	R-sq	R-sq(adj)	R-sq(pred)
0.597934	47.26%	43.09%	33.52%

**Table 9:** Coefficients of the regression.

Term	Coef	SE Coef	T-Value	P-Value	VIF
Constant	0.693	0.825	0.84	0.403	
POR	−0.254	0.109	−2.33	0.022	1.52
TAS	0.042	0.127	0.33	0.744	1.69
QOW	0.190	0.140	1.35	0.179	1.50
EJY	0.314	0.138	2.28	0.026	1.62
SIF	0.264	0.123	2.15	0.035	1.33
OAD	0.170	0.068	2.48	0.015	1.10

## 2 Experimental design, materials, and methods

Institut Teknologi Sepuluh Nopember (ITS) was selected in East Java, Indonesia. The total number of students is 20448 students. Using Yamane's formula of Yamane [2] with 95% confidence level, three hundred and ninety-three students were selected as the respondents. The students were selected randomly by sampling the student registration number, assuming that the students are homogeneous on their perception and understanding about online shopping behaviour. Furthermore, the students were asked to fill in the online questionnaire through the provided link. The data presented in this paper is focused only on the students who experienced online shopping. Among those 393 students, there were 83 students who did online shopping. The research was conducted according to and complies with all regulations established in the ethical guidelines by the ITS Research Ethics Committee in the “code of ethics”. All participants provided written informed consent.

**Fig. 3:**
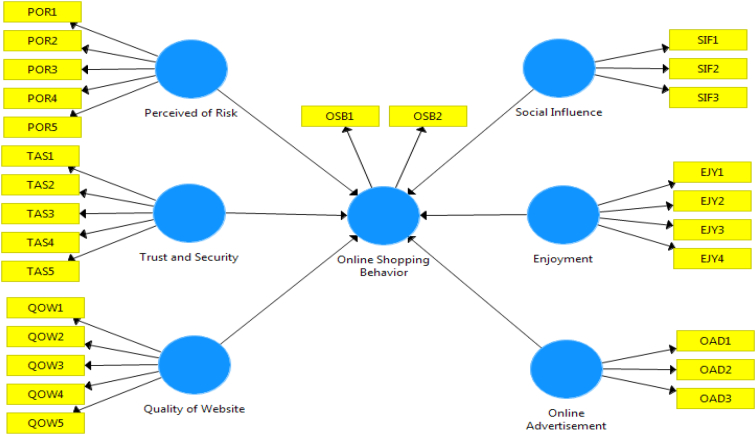
Conceptual framework of the variables.

The questionnaire was made following the conceptual framework of Moshref et al. [3], as can be seen in Fig. 3. The questionnaire comprises students characteristics and their perceptions on online shopping behaviour with a Likert scale (strongly disagree (1) – strongly agree (5)). The perception variables were measured for online shopping behaviour (OSB) as the response and six predictiors, i.e. perception of risk (POR), trust and security (TAS), enjoyment (EJY), quality of website (QOW), online advertisment (OAD), and social influence (SIF). The list of questions (indicators) for each variable can be seen in the labels of the SPSS file for the corresponding variable. Mean of each perception variables are given in the data for the sake of building regression model. Validity and reliability of the data are confirmed by the test, as can be seen in Table 10 and Table 11, respectively. All reliability indicators are greater than 0.5 indicating that the data is reliable.

**Table 10:** Validity test.

	EJY	OAD	OSB	POR	QOW	SIF	TAS
EJY1	0.797	0.159	0.493	−0.148	0.419	0.355	0.146
EJY2	0.878	0.283	0.584	−0.356	0.422	0.439	0.367
EJY3	0.765	0.157	0.397	−0.267	0.381	0.366	0.237
EJY4	0.775	0.143	0.377	−0.319	0.471	0.340	0.482
OAD1	0.246	0.900	0.268	−0.011	0.196	0.233	0.069
OAD2	0.147	0.918	0.301	−0.102	0.272	0.248	0.181
OAD3	0.249	0.869	0.341	−0.090	0.148	0.198	0.184
OSB1	0.597	0.218	0.895	−0.424	0.493	0.542	0.409
OSB2	0.375	0.393	0.799	−0.324	0.29	0.355	0.310
POR1	−0.248	−0.033	−0.352	0.729	−0.164	−0.072	−0.350
POR2	−0.135	−0.064	−0.169	0.635	−0.025	0.002	−0.390
POR3	−0.120	−0.134	−0.212	0.764	−0.089	−0.054	−0.530
POR4	−0.276	−0.173	−0.383	0.685	−0.060	−0.102	−0.355
POR5	−0.311	0.108	−0.337	0.699	−0.210	−0.262	−0.452
QOW1	0.335	0.135	0.316	−0.188	0.799	0.327	0.289
QOW2	0.434	0.344	0.503	−0.168	0.834	0.429	0.369
QOW3	0.426	0.149	0.389	−0.120	0.806	0.374	0.301
QOW4	0.396	0.099	0.234	0.017	0.664	0.233	0.058
QOW5	0.421	0.043	0.301	−0.154	0.739	0.410	0.230
SIF1	0.220	0.026	0.352	−0.113	0.163	0.722	0.070
SIF2	0.205	0.245	0.287	−0.095	0.142	0.715	0.273
SIF3	0.500	0.254	0.471	−0.127	0.584	0.705	0.311
TAS1	0.349	0.173	0.471	−0.572	0.237	0.342	0.847
TAS2	0.294	0.056	0.366	−0.489	0.230	0.134	0.853
TAS3	0.215	0.225	0.207	−0.28	0.387	0.241	0.738
TAS4	0.36	0.137	0.315	−0.505	0.314	0.222	0.834
TAS5	0.268	0.118	0.294	−0.399	0.344	0.334	0.792

**Table 11:** Reliability test.

	Cronbach's Alpha	rho_A	Composite Reliability	Average Variance Extracted (AVE)
EJY	0.819	0.847	0.880	0.648
OAD	0.878	0.886	0.924	0.803
OSB	0.617	0.654	0.836	0.719
POR	0.754	0.753	0.830	0.496
QOW	0.830	0.874	0.879	0.594
SIF	0.543	0.518	0.757	0.510
TAS	0.876	0.915	0.907	0.662

## 3 Policy implications

The data revealed that the students’ online shopping behaviour is significantly influenced by the perception of risk (POR), enjoyment (EJY), social influence (SIF) and online advertisment (OAD). Considering the fact that students are a potential market, the online marketplace should put more focus on those variables. Market segmentation is also important to formulate an efficient marketing strategy. To this end, the data presented in this article is useful for further comprehensive analysis.
