# Supplementary material for: Survey data on students’ online shopping behaviour: A focus on selected university students in Indonesia
Source: Data Brief. 2020 Jan 2;29:105073. doi: 10.1016/j.dib.2019.105073 (PMC6965723; doi:10.1016/j.dib.2019.105073)
Supplement: Multimedia component 1 [file mmc1.pdf]

Questionnaire

Dear Students,

We are conducting a study about students' online shopping behaviour. You are selected as one of our respondents. Please do not mind to fill this questionnaire based on your situation.

Thank you in advance for your support and participation.

Best regards

Team

Screening
Are you a student of Institut Teknologi Sepuluh Nopember and have done transactions using online shopping sites in the last 3 months?

a. Yes
b. No

Identity of Respondent

1. Name :

2. NRP :

3. Age (in years) :

4. Sex of respondent :
   a. Male
   b. Female

5. Allowance (per month) :

6. Semester :

7. Internet usage per day (in hours) :

8. Frequency of online shopping transactions per month :

9. Favorite online shopping site :
   a. Tokopedia
   b. Bukalapak
   c. Blibli.com
   d. Lazada
   e. Elevenia
   f. OLX

g. Blanja.com
h. BerryBenka
i. Shopee
j. Others

10. Favorite type of products :
    a. New
    b. Secondhand
    c. Others

11. Frequently purchased products :
    a. New
    b. Secondhand

12. Type of provider :
    a. Telkomsel
    b. Indosat
    c. XL
    d. Axis
    e. 3
    f. Smartfren
    g. Others

13. Frequently purchased type of products :
    a. Accessories
    b. Electronic
    c. Fashion
    d. Hobbies
    e. Services
    f. Home furniture
    g. Skincare
    h. Voucher

14. Price range :
    a. < Rp. 500.000
    b. Rp. 500.000-1.000.000
    c. > Rp. 1.000.000

15. Frequently used gadget for online shopping :
    a. Laptop
    b. Personal Computer (PC)
    c. Handphone
    d. Tablet

16. Type of payment
    a. Cash on Delivery
    b. ATM transfer

c. Credit card
d. Debit card
e. E-banking

Perceived Risk

1. Consumer is in doubt about online shopping due to the high risk of getting a defective product :
   a. Strongly disagree
   b. Disagree
   c. Neutral
   d. Agree
   e. Strongly agree

2. Consumer thinks that there will be difficulty in solving problems that arise through online shopping (such as exchanged product) :
   a. Strongly disagree
   b. Disagree
   c. Neutral
   d. Agree
   e. Strongly agree

3. Consumer might receive a defect product :
   a. Strongly disagree
   b. Disagree
   c. Neutral
   d. Agree
   e. Strongly agree

4. It s difficult to assess the product quality online :
   a. Strongly disagree
   b. Disagree
   c. Neutral
   d. Agree
   e. Strongly agree

5. Consumer might not receive the product ordered :
   a. Strongly disagree
   b. Disagree
   c. Neutral
   d. Agree
   e. Strongly agree

Online Advertisement

1. Online advertising influences the consumer to buy a new product :
   a. Strongly disagree

b. Disagree
c. Neutral
d. Agree
e. Strongly agree

2. Online advertising influences the consumer to try another type of product :
   a. Strongly disagree
   b. Disagree
   c. Neutral
   d. Agree
   e. Strongly agree

3. Online advertising influences the decision to change to another brand :
   a. Strongly disagree
   b. Disagree
   c. Neutral
   d. Agree
   e. Strongly agree

Trust and Security

1. Consumer feels convenience with online shopping :
   a. Strongly disagree
   b. Disagree
   c. Neutral
   d. Agree
   e. Strongly agree

2. Consumer trusts the online shopping website :
   a. Strongly disagree
   b. Disagree
   c. Neutral
   d. Agree
   e. Strongly agree

3. Customer feels that privacy is maintained when doing online shopping :
   a. Strongly disagree
   b. Disagree
   c. Neutral
   d. Agree
   e. Strongly agree

4. Online shopping website has good safety features :
   a. Strongly disagree
   b. Disagree
   c. Neutral
   d. Agree

e. Strongly agree

5. Online shopping website has a good reputation :
    a. Strongly disagree
    b. Disagree
    c. Neutral
    d. Agree
    e. Strongly agree

Social influence

1. People around the customer frequently do online shopping :
    a. Strongly disagree
    b. Disagree
    c. Neutral
    d. Agree
    e. Strongly agree

2. People around the customer recommend online shopping over traditional shopping :
    a. Strongly disagree
    b. Disagree
    c. Neutral
    d. Agree
    e. Strongly agree

3. Technological development influences the customer to shop online :
    a. Strongly disagree
    b. Disagree
    c. Neutral
    d. Agree
    e. Strongly agree

Quality of Website

1. Consumer buys from online shop if it is visually interesting and has a well-organized appearance :
    a. Strongly disagree
    b. Disagree
    c. Neutral
    d. Agree
    e. Strongly agree

2. Consumer buys from online shop if the navigation flow is user friendly :
    a. Strongly disagree
    b. Disagree
    c. Neutral

d. Agree
e. Strongly agree

3. Consumer buys from online shop if it is easy to understand the website and its provided information :
    a. Strongly disagree
    b. Disagree
    c. Neutral
    d. Agree
    e. Strongly agree

4. Consumer buys from online shop if it has free and easy shipment order fees and transaction procedures :
    a. Strongly disagree
    b. Disagree
    c. Neutral
    d. Agree
    e. Strongly agree

5. Consumer buys from online shop if it gives complete information about the products :
    a. Strongly disagree
    b. Disagree
    c. Neutral
    d. Agree
    e. Strongly agree

Enjoyment

1. Online shopping saves time to shop :
    a. Strongly disagree
    b. Disagree
    c. Neutral
    d. Agree
    e. Strongly agree

2. Online shopping makes it easy to purchase goods :
    a. Strongly disagree
    b. Disagree
    c. Neutral
    d. Agree
    e. Strongly agree

3. Information and facility make it easy to compare and purchase goods :
    a. Strongly disagree
    b. Disagree

c. Neutral
d. Agree
e. Strongly agree

4. Online shopping is easy to be carried out in general :
   a. Strongly disagree
   b. Disagree
   c. Neutral
   d. Agree
   e. Strongly agree

Online shopping behaviour

1. Customer will repeat the online shopping :
   a. Strongly disagree
   b. Disagree
   c. Neutral
   d. Agree
   e. Strongly agree

2. Consumer prefers online shopping over conventional shopping :
   a. Strongly disagree
   b. Disagree
   c. Neutral
   d. Agree
   e. Strongly agree
